# Media Awareness and Screen Time Reduction in Children, Youth or Families: A Systematic Literature Review

**DOI:** 10.1007/s10578-021-01281-9

**Published:** 2021-12-02

**Authors:** Hanno Krafft, Katja Boehm, Silke Schwarz, Michael Eichinger, Arndt Büssing, David Martin

**Affiliations:** 1 Chair of Medical Theory, Integrative and Anthroposophic Medicine, Witten/Herdecke University, Alfred-Herrhausen-Straße 50, 58448 Witten, Germany; 2 Medical Faculty Mannheim, Heidelberg University, Heidelberg, Germany; 3 Department of Pediatrics, Tübingen University, Tübingen, Germany

**Keywords:** Screen time, Child, Digital media, Intervention, Review

## Abstract

Excessive use of screen media is a global public health issue and especially extensive screen exposure during very early childhood. This review was conducted in order to update previous reviews on the effectiveness of interventions to reduce screen time. An electronic literature search was carried out in MEDLINE, COCHRANE LIBRARY and CINAHL for articles indexed from June 2011 until October 2019. The search identified 933 publications of which 11 publications were included in this review. There are studies showing interventions with a positive influence on reduction of screen time and the participants’ awareness and behavior concerning the use of screen media, as well as studies without such effects. No intervention was identified to be superior. This warrants further investigation of potentially effective combinations of intervention components and long-term follow-up.

## Introduction

### Rationale

Excessive use of digital screen media (screen time) is a global public health issue associated with adverse mental and physical health outcomes, especially for children. During the past few years children of all ages have not only obtained access to the possibilities of traditional screens like TV but additionally have access to new screen technologies like, for instance, computers, tablets, smartphones and gaming consoles [1, 2]. Studies show that, as they are getting older, children and adolescents spend more time in front of screen media [3], and that the time children use screen media for the first time, is happening earlier [2, 4]. Numerous studies have shown that extensive screen exposure during very early childhood can be harmful: for cognitive development [5–7], social competences [8, 9], mental health [9, 10] and physical wellbeing [1, 11]. In their review of effective strategies for reducing screen time among young children from 2012, Schmidt et al. [12] put forward some research priorities and recommendations for the planning of an intervention based on gaps in the current literature. Developing interventions that are scalable to children, adolescence and adults needs multifaceted programs with different components and such components need to be evaluated for their single and combined effectiveness. The present review builds the background for the best possible planning of interventions.

### Objectives

To our knowledge, only one systematic review has examined intervention strategies to reduce screen time among children from birth to 12 years of age. In 2012 Schmidt et al. conducted a systematic review of 7 electronic databases to June 2011, using the terms “intervention” and “television”, “media” or “screen time”. They identified 47 out of 144 peer‐reviewed intervention studies that reported frequencies of TV viewing or screen‐media use in children were included. Significant reductions in TV viewing or screen‐media use were achieved in 29 studies. Interventions utilizing electronic TV monitoring devices, contingent feedback systems, and clinic‐based counseling were most effective. Schmidt et al. found several research gaps, including a relative paucity of studies targeting young children or minorities, limited long‐term (> 6 month) follow‐up data, and few targeting removing TVs from children’s bedrooms [12].

Because of the rapid development in screen media, especially smartphones and handheld devices, we decided to update this systematic review by reviewing studies published between June 2011 and October 2019, to see whether there are new studies on this topic that might be helpful for the development of interventions generating media awareness and attempts to reduce screen time.

## Methods

**Fig. 1:**
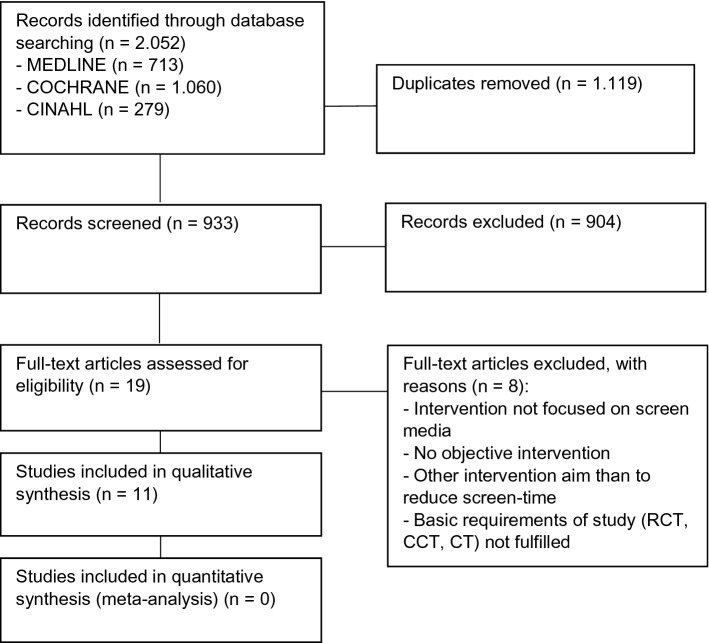
Flow diagram of study selection

Our systematic review was conducted in accordance with the recommendations of the PRISMA (Preferred Reporting Items for Systematic Reviews and Meta-Analyzes) statement [13] (Fig. 1).

### Information Sources and Search

An electronic literature search was carried out in the databases MEDLINE, COCHRANE LIBRARY and CINAHL. All articles indexed between June 2011 and October 2019 were considered for eligibility. Searches were carried out with English keywords related to screen-media, intervention and study design:

- media, television, TV, mobile, handheld, tablet, smartphone, gaming, game, computer, electronic device, video, screen
- intervention, education, information, behavior, change, reduce,
- randomized controlled trial (RCT), controlled clinical trial (CCT), clinical trial (CT)

**Fig. 2:**
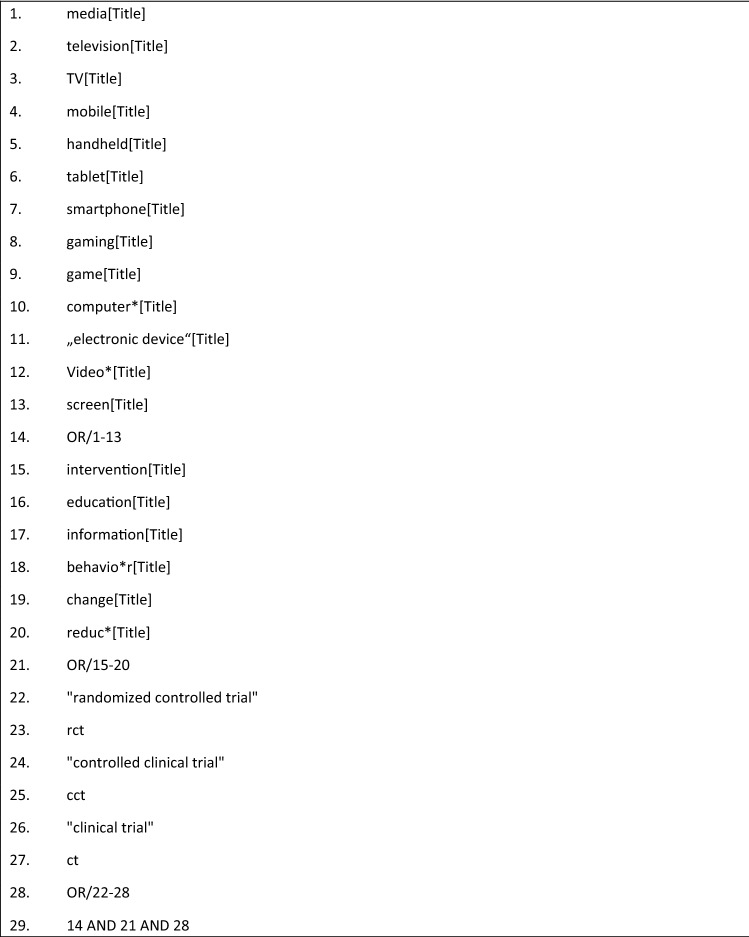
Search strategy for the MEDLINE database

The complete electronic search strategy for the MEDLINE database, including any limits used, is provided in Fig. 2 in the Appendix. Comparable search strategies were used for the other databases.

### Study Selection and Eligibility Criteria

Studies were independently considered for eligibility by two reviewers. The following eligibility criteria were used: (1) Intervention focused on screen media, (2) There was an objective intervention, (3) Intervention aimed to reduce screen time, (4) Basic requirements of a study (RCT, CCT, CT) were fulfilled. Exclusion criteria were (1) Full text not available in English language, (2) Participants older than 18 years. Predefined data abstraction forms were used to collect relevant data for all eligible studies (Table 1).

**Table 1:** Study characteristics

Author, year, design	Sample size, participants, age	Media	Intervention	Duration	Outcome	Results
Bandeira et al. 2019 [17], RCT	1085 students, aged 11–13 and 14–17 years	TV/video games/computer	Teacher training, support material for teachers, environmental opportunities to encourage physical activity, health education messages in schools via posters etc	4 months	Student reported screen time on weekdays and weekends	No significant differences between intervention and control groups for reduction on screen time (boys: 0.105 h/day, 95% CI: − 0.184 to 0.393, p = 0.477; girls: − 0.065 h/day, 95% CI: − 0.383 to 0.252, p = 0.686) and age groups (11–13 years: − 0.046 h/day, 95% CI: − 0.630 to 0.538, p = 0.878; 14–17 years: 0.193 h/day, 95% CI: − 0.077 to 0.464, p = 0.162)
Smith et al., 2017 [18], RCT	361 boys, mean age, 12.7 ± 0.5 years	Screen time in general	Smartphone App "ActiveTeen Leaders Avoiding Screen time” (ATLAS)	18 months	Screen time reported each day of the week	Significant intervention effect was observed for recreational screen time at 8-months (CI = -33 min/d; p = 0.001), which was sustained at 18-months (CI = -27 min/d; p = 0.007)
Babic et al., 2016 [19], RCT	322 students, age unknown	TV/video/DVD/computer/tablet/smartphone	Interactive seminar, informational and motivational messages via preferred social media and messaging systems	6 months	Adolescents reported mean daily screen time	Significant reductions in screen time were observed in both groups from baseline to posttest (Intervention = -50.5 min/d, p < 0.001; control = -29.2 min/d, p = 0.030) The adjusted between-group difference was not statistically significant (mean = -21.3 min/d; p = 0.255)
Mendoza et al., 2016 [20], RCT	211 children, aged 3–5 years	TV	Fit 5 Kids (F5K) TV reduction curriculum	8 weeks	TV viewing screen time	Significant relative difference for the decrease in mean daily TV viewing minutes − 25.3 (95% CI = − 45.2, − 5.4) for the intervention versus the control group (p = 0.01)
Yilmaz et al., 2015 [21], RCT	363 children, mean age 3.5 ± 1.25 years	TV/video games/computer	Printed materials, interactive CD’s and 1 counselling call	9 months	Length of screen time of children for 1 week	Significant reduction from baseline in screen time for the control and intervention groups over time (93.96 ± 18.84/21.15 ± 6.12/t = 50.1, p < 0.001)
Andrade et al., 2015 [22], RCT	1370 adolescents, mean age 12.8 ± 0.8 years	TV/video games/computer	Individual and environmental oriented strategies i.e. manuscript, textbook, parental workshops	28 months	Adolescents reported screen time on week- and weekend days	While there was partial reduction of screen time in favor to the control group, no constant reduction in screen time was observed in the intervention group over the whole intervention period
Lubans et al., 2014 [23], RCT	361 adolescent boys, mean age, 12.7 ± 0.5 years	Screen time in general	Smartphone App "ActiveTeen Leaders Avoiding Screen time” (ATLAS)	20 weeks	Intention to reduce screen time	Participants’ intentions to limit their recreational screen time in percent agreement (mean D 3.95 ± 1.07) were high following the completion of the program
Maddison et al., 2014 [24], RCT	251 children, aged 9–12 years	TV/video/computer	Face-to-face counseling of parent/caregiver/child, activity packages, online support via a website, monthly newsletters	20 weeks	Screen-based sedentary time	No significant differences in screen-based sedentary behavior in intervention and control groups (95% CI − 33, − 73,7 p = 0.11)
Hesketh et al., 2014 [25], RCT	542 children, ∼3-months old	TV	Melbourne InFANT Program (Material and sources to provide knowledge, skills and strategies to promote healthy eating and active play)	17 months	TV viewing screen time	The intervention reduced children’s television viewing time (-14.62; 95% CI − 28.02, − 1.24)
Birken et al., 2012 [26], RCT	351 children, 3-years-old	TV/video games/computer	10-min behavioral counseling intervention by trained study personal directly after the health maintenance visit, which included information on the health impact of screen time in children and provided strategies to decrease screen time	12 months	Parent-reported screen time of children	No significant differences in mean total weekday minutes of screen time (60 min, IQR: 35–120 vs. 65 min, IQR: 35–120; p = 0.68), or mean total weekend day minutes of screen time (80 min, IQR: 45–130 vs. 90 min, IQR: 60–120, p = 0.33), between the intervention and control groups
Mendelsohn et al., 2011 [27], RCT	410 families with a child, mean age, 6.9 ± 1,3 months	TV/video games/computer	Videotaping of mother–child interaction followed by review with the child development specialist, provision of learning materials, provision of parenting pamphlets, newsletters, learning materials, and parent-completed developmental questionnaires	6 months	Total daily exposure for the child during a 24-h period	Differences were found across the 2 intervention group and 1 control groups for daily duration of media exposure (min/d 131.6 ± 118.7, 151.2 ± 116.7), 155.4 ± 138.7, p = 0.03). Further children in 1 of 2 intervention groups have been first exposed to media approximately half a month later than children in control groups (p = 0.01)

*CI* confidence interval, *IQR* interquartile range, *SD* standard deviation

### Risk of Bias in Individual Studies

The methodological quality of all studies was assessed using the PEDro scale which covers both internal and external validity [14]. The PEDro scale was originally developed to evaluate the titles indexed in the PEDro database and to indicate to the user, whether the randomized clinical trials are internally valid and provide sufficient statistical information to interpret the results. The PEDro scale, which is based on the Delphi list of Verhagen and his colleagues [15], consists of a total of 11 items that are answered with “yes” or “no”. Items 2–11 focus on different aspects of internal validity. All positive answers to items 2–11 are summed up and give the PEDro score (range: 0–10). Studies with a PEDro score of ≥ 6 points are considered “high quality”, and studies with a PEDro score of ≤ 5 points are rated as “low quality” [16] Item 1 refers to the specification of eligibility criteria of study participants (external validity) and is not taken into account in the calculation of the PEDro score [14].

### Risk of Bias Within Studies

The methodological quality of the included studies ranged from 4 to 8 points on the PEDro scale and can be rated as good overall (median: 6 points, Table 2). None of the 11 studies achieved a perfect score. However, 9 studies obtained PEDro scores of ≥ 6 and can be categorized as high-quality studies. The unconcealed allocation as well as the missing blinding of subjects, therapists and assessors were the most frequent methodological deficiencies. The internal validity of the results is therefore limited (see Table 2).

**Table 2:** Risk of bias within studies

Author	Eligibility criteria specified^a^	Subjects randomly allocated^b^	Concealed allocation^c^	Similar baseline^d^	Subjectsblinded^e^	Therapists blinded^f^	Assessors blinded^g^	Measures obtained from 85% of subjects^h^	Intention to treat analysis^i^	Between-group comparison^j^	Point and variability measures^k^	Overall quality (PEDro-Score)
Bandeira et al. [17]	y	1	0	1	0	0	0	1	1	1	1	6/10
Smith et al. [18]	n	1	0	1	0	0	0	1	1	1	1	5/10
Babic et al. [19]	y	1	1	1	0	0	0	1	1	1	1	7/10
Mendoza et al. [20]	y	1	1	1	0	0	0	0	1	1	1	6/10
Yilmaz et al. [21]	n	1	0	1	0	0	1	1	0	1	1	6/10
Andrade et al. [22]	y	1	0	1	0	0	1	0	1	1	1	6/10
Lubans et al. [23]	y	1	0	1	0	0	0	0	1	1	1	5/10
Maddison et al. [24]	y	1	1	1	0	0	0	1	1	1	1	7/10
Hesketh et al. [25]	n	1	0	1	0	0	0	0	0	1	1	4/10
Birken et al. [26]	y	1	1	1	0	1	1	0	1	1	1	8/10
Mendelsohn et al. [27]	y	1	1	1	0	0	0	0	1	1	1	6/10

^a^Eligibility criteria were specified

^b^Subjects were randomly allocated to groups (in a crossover study, subjects were randomly allocated an order in which treatments were received)

^c^The allocation was concealed until the end of recruitment

^d^The groups were similar at baseline regarding important prognostic factors

^e^All subjects were blinded

^f^All persons administering the intervention were blinded

^g^All assessors who measured at least one key outcome were blinded

^h^Data for at least one key outcome were obtained from more than 85% of the subjects initially allocated to both groups

^i^All subjects for whom outcome measures were available, data for at least one key outcome was analyzed by an intention to treat analysis

^j^The results of between-group comparisons were reported for at least one key outcome

^k^Both point estimates and measures of uncertainty were reported for at least one key outcome

## Results

### Study Selection

The electronic literature search identified 2.052 publications, of which 933 articles remained after the exclusion of duplicates. In the title and abstract screening 914 publications were excluded as not relevant. After assessing the full texts of the remaining articles for eligibility another 8 publications were excluded. A total of 11 publications were included in the qualitative synthesis [17–27] (Fig. 1).

### Study Characteristics and Respective Results

In a recent RCT Bandeira et al. investigated 1085 students, aged 11–17 years, regarding their screen time including TV, video games and computer on weekdays and weekends. The intervention had a duration of 4 months and included teacher trainings, the provision of support material for teachers, environmental opportunities to encourage physical activity and health education messages in schools via posters. Screen time was evaluated using an adapted questionnaire. No significant differences for reduction of screen time between the intervention group and the control group, which received no intervention were found [17]. In another RCT published in 2017 Smith et al. examined 361 boys regarding their overall screen time during each day of the week. The intervention was the smartphone application “Active Teen Leaders Avoiding Screen time” (ATLAS), which was designed to promote physical activity and reduce screen-time in adolescent boys considered at risk of obesity. A significant intervention effect was observed for recreational screen time at 8-months which was sustained at 18-months [18]. In a further RCT published in 2016 Babic et al. investigated 322 students of unknown age regarding their mean daily screen time including the use of TVs, videos, DVDs, computers, tablets and smartphones. The intervention with a duration of 6 months consisted of interactive seminars and informational as well as motivational messages via preferred social media and messaging systems. Reductions in screen time were observed in both groups from baseline to end line. However, there was no evidence for an adjusted between-group difference at end line [19]. Mendoza et al. published a RCT where the TV viewing in minutes/day was investigated in 211 children aged 3–5 years. The intervention was called the Fit 5 Kids (F5K) TV reduction curriculum and lasted for 8 weeks. Significant relative difference for the decrease in mean daily TV viewing minutes for the intervention versus the control group, which received no intervention, were observed [20]. Yilmaz et al. investigated 363 children, aged 3.5 years in a RCT regarding the length of screen time (TV/video games/computer) for 1 week. The intervention consisted of printed materials, interactive CDs and 1 counselling call over 9 months. Significant reduction from baseline in screen time were observed for the control as well as the intervention groups over time [21]. In another RCT, Andrade et al. reported the number of hours spent of screen time of TV/video games/computer on week and weekend days for 1370 adolescents. The intervention with a duration of 28 months consisted of individual and environmental strategies (i.e. manuscript, textbook, parental workshops). While there were partial reduction of screen time in favor to the control group, no constant reduction in screen time was observed in the intervention group over the whole intervention period [22]. In another RCT published by Lubans et al. in 2014 the smartphone app “ActiveTeen Leaders Avoiding Screen time” (ATLAS) was also used as an intervention for 20 weeks with 361 adolescent boys and their intention to reduce screen time in general was examined. Participants’ intentions to limit their recreational screen time in percent agreement were high following the completion of the program [23]. Another RCT by Maddison et al. investigated the screen-based sedentary time (min/d) spend on TV/video/computer in 251 children aged 9–12 years. For 20 weeks multiple face-to-face counseling of parent/caregiver/child, activity packages, online support via a website and a monthly newsletter was provided as intervention. No significant differences in screen-based sedentary behavior in neither the intervention nor control groups were observed [24]. In 2014 Hesketh et al. reported about a RCT in which TV viewing times of 542 3-months-old children were examined. The intervention was the Melbourne InFANT Program (Material and sources to provide knowledge, skills and strategies to promote healthy eating and active play) which was carried out for 17 months. The intervention reduced children’s television viewing time [25]. Birken et al. published a RCT regarding parent-reported time 3-years-old children spent on TV/video games/computer. The intervention in 351 children over 12 months was a 10-min behavioral counseling by trained study personal directly after the health maintenance visit, which included information on the health impact of screen time in children and provided strategies to decrease screen time. No significant differences in mean total weekday minutes of screen time, or mean total weekend day minutes of screen were observed between the intervention and control groups [26]. Mendelsohn et al. carried out a RCT investigating 410 families with a child mean age of 6.9 months and reported the daily exposure to TV/video games/computer in minutes during a 24-h period for each child. As intervention videotaping of mother–child interaction followed by review with the child development specialist, provision of learning materials, provision of parenting pamphlets, newsletters, learning materials, and parent-completed developmental questionnaires were carried out over 6 months. Differences were found across groups for daily duration of media exposure and children in 1 of 2 intervention groups had first been exposed to media approximately half a month later than children in control groups [27]. For an overview of all study characteristics with detailed statistical values see Table 1.

### Overall Summarized Results of Studies

In 6 studies there was evidence for a reduction in screen time [18–21, 25, 27]. Moreover, in 1 study participants’ intentions to limit their recreational screen time were high following the completion of the program [23] and in another study children of 1 intervention group were first exposed to media approximately half a month later than children in the control groups [27]. No significant differences between intervention and control groups for reduction of screen time were found in four studies [17, 22, 24, 26]. For an overview of all individual results, see Table 1.

Comparing the interventions in the studies with regard to their components, the following classification can be made: components that rely on personal knowledge transfer like school curriculums [20] and teacher training [17] or face-to-face counseling [24, 26, 27], counselling calls [21] and workshops [22]; components that impart knowledge via printed information materials (i.e. manuscripts, textbooks, posters) [17, 21, 22, 25, 27]; Digital or online components like interactive seminars [19, 21], newsletters [24, 27], information via websites [24], smartphone App [18, 23] or social media and messaging systems [19].

The kind of screen media which was targeted by the interventions was also different across the included studies: seven studies defined screen media as TV, video (games) and computer [17, 19, 21, 22, 24, 26, 27]; two studies only named TV [20, 25] and two studies try to reduce screen time in general [18, 23]; one study focused on tablets and smartphones beside TV, video and computer [19].

## Discussion

### Summary of Evidence

The majority of studies showed that different interventions can have an effect on screen time [18–21, 25, 27] or at least have a positive influence on the participants’ awareness and behavior concerning the use of screen media [23, 27]. These results are consistent with the existing review of Schmidt et al. [12]. It is hard to speculate on why there are also studies that show no significant differences between intervention and control groups [17, 22, 24, 26] since the included participants, applied interventions as well as measurements were similar to other studies. It is possible that there are particularly effective combinations of interventions components, but from the existing data, no conclusions can be drawn as to which intervention component is more effective or superior. Especially since most interventions use multiple components and these components were not evaluated separately. Short interventions focusing solely on reducing screen time may not be effective in preschool children [26] but focusing on screen time behavior in combination with other health behaviors might result in a greater effect on screen time [28]. Another issue is the fact that most adolescents increased their screen time again [22] after interventions (in terms of a ‘rebound-effect’), which suggests that long-term interventions may be necessary for achieving long-lasting awareness and behavior changes. Only one study [27] included expectant mothers, parents of newborns and infants. Because the studies all related to different media, no general statement can be made about the effect of the interventions. Especially since most of the studies focused on conventional screen media such as TV and computers, and only one study included smartphones and tablets [19].

### Limitations

Firstly, an electronic literature search inherently contains limitations: only three databases with terms in English were searched, meaning that articles that are not indexed in those databases or are published in any other language were not included and therefore, the search strategy may not have identified all publications. The search strategy only looked for randomized controlled trials, controlled clinical trials and clinical trials. Alternative interventional study designs, such as quasi-experimental designs, were omitted. On the basis of title and abstract and according to the a priori defined inclusion and exclusion criteria, a high number of publications were excluded. There were situations in which the decision to include or exclude a questionnaire was unclear, due to incomplete or ambiguous information. Here an attempt was made to make a scientifically valid decision shared between at least two of the authors. It is nevertheless possible that relevant publications were not considered. Due to study heterogeneity in terms of methodologies, outcomes and measurement instruments, carrying out a meta-analysis was not possible. Although we decided to analyze the studies regarding their effect in a qualitative comparison, the different interventions across studies made it difficult to compare findings. When a significant reduction of screen time was reported, it was often unclear which specific media was reduced. In addition, in a large number of articles the interventions or the control groups were not described very detailed and reproducibly. There was only 1 study which assessed the intention to reduce screen time. The motivation of adolescents to reduce it might be a crucial point; however, it was not assessed in the studies and thus the relevance of reported outcomes (minutes per day) is difficult to judge. None of the studies were conducted in Germany and therefore results cannot be transferred without restriction to German-speaking countries due to possible transcultural differences. Given the fact that nearly all experts worldwide agree that children spend too much time in front of screens, the fact that we only found 11 interventional studies between 2012 and 2019, and none in Germany, is rather disconcerting. Given the paucity of long-term, early onset trials, our group is planning to undertake such trials and would like to extend invitations for collaboration to all interested.

## Conclusions

The fact that there are studies showing interventions with significant effect or a positive influence on reduction of screen time and the participants’ awareness and behavior concerning the use of screen media, as well as studies with no significant differences between intervention and control groups, indicates that particularly effective combinations of intervention components must be further investigated. The content and duration of identified interventions was highly heterogeneous, and thus the study findings are difficult to compare. Short interventions may not be effective since most participants increased their screen time again following the interventions. Future research should explore the effects of long-term interventions, generating media awareness and attempts to reduce screen time from birth to adulthood in prospective longitudinal studies need to address expectant mothers, parents, children and adolescents in age-specific ways. Further investigation should also increasingly focus on new digital screen media like smartphone and tablets.

## Summary

Excessive use of digital screen media (screen time) is a global public health issue associated with adverse mental and physical health outcomes, especially for children. During the past few years children of all ages have not only obtained access to the possibilities of traditional screens like TV but additionally have access to new screen technologies. Only one systematic review from 2012 has examined intervention strategies to reduce screen time among children from birth to 12 years of age. Because of the rapid development in screen media, especially smartphones and handheld devices, we decided to update this systematic review by reviewing studies published between June 2011 and October 2019. An electronic literature search with English keywords related to screen-media, intervention and study design was carried out in MEDLINE, COCHRANE LIBRARY and CINAHL. The search identified 2052 publications of which 11 publications were included in the qualitative synthesis. The methodological quality of the included studies can be rated as good overall on the PEDro scale (median: 6 points). The internal validity of the results is therefore limited. The majority of studies showed that different interventions can have an effect on screen time or at least have a positive influence on the participants’ awareness and behavior concerning the use of screen media. These results are consistent with the existing review from 2012. Short interventions focusing solely on reducing screen time may not be effective in preschool children but focusing on screen time behavior in combination with other health behaviors might result in a greater effect on screen time. Another issue is the fact that most adolescents increased their screen time again after interventions, which suggests that long-term interventions may be necessary for achieving long-lasting awareness and behavior changes. Because the studies all related to different media, no general statement can be made about the effect of the interventions. The fact that there are studies showing interventions with significant effect or a positive influence on reduction of screen time and the participants’ awareness and behavior concerning the use of screen media, as well as studies with no significant differences between intervention and control groups, indicates that particularly effective combinations of intervention components must be further investigated, including new digital screen media like smartphone and tablets.

## Data Availability

Not applicable.

## Appendix

See Fig. 2.

## References

[1] Wolf C, Wolf S, Weiss M, Nino G (2018). Children’s environmental health in the digital era: understanding early screen exposure as a preventable risk factor for obesity and sleep disorders. Children.

[2] Madigan S, Racine N, Tough S (2019). Prevalence of preschoolers meeting vs exceeding screen time guidelines. JAMA Pediatr.

[3] Must A, Tybor DJ (2005). Physical activity and sedentary behavior: a review of longitudinal studies of weight and adiposity in youth. Int J Obes.

[4] Anderson DR, Pempek TA (2005). Television and very young children. Am Behav Sci.

[5] Domingues-Montanari S (2017). Clinical and psychological effects of excessive screen time on children. J Paediatr Child Health.

[6] Tomopoulos S, Dreyer BP, Berkule S, Fierman AH, Brockmeyer C, Mendelsohn AL (2010). Infant media exposure and toddler development. Arch Pediatr Adolesc Med.

[7] Hutton JS, Dudley J, Horowitz-Kraus T, DeWitt T, Holland SK (2019). Associations between screen-based media use and brain white matter integrity in preschool-aged children. JAMA pediatrics.

[8] Griffiths M (1997) Friendship and social development in children and adolescents: the impact of electronic technology 14

[9] McDonald SW, Kehler HL, Tough SC (2018). Risk factors for delayed social-emotional development and behavior problems at age two: Results from the All Our Babies/Families (AOB/F) cohort. Health Sci Rep.

[10] Babic MJ, Smith JJ, Morgan PJ, Eather N, Plotnikoff RC, Lubans DR (2017). Longitudinal associations between changes in screen-time and mental health outcomes in adolescents. Ment Health Phys Act.

[11] Twenge JM, Campbell WK (2018). Associations between screen time and lower psychological well-being among children and adolescents: evidence from a population-based study. Prevent Med Rep.

[12] Schmidt ME, Haines J, O’Brien A, McDonald J, Price S, Sherry B, Taveras EM (2012). Systematic review of effective strategies for reducing screen time among young children. Obesity.

[13] Moher D, Liberati A, Tetzlaff J, Altman DG, PRISMA Group (2009). Preferred reporting items for systematic reviews and meta-analyses: the PRISMA statement. PLoS Med.

[14] Sherrington C, Herbert RD, Maher CG, Moseley AM (2000). PEDro. A database of randomized trials and systematic reviews in physiotherapy. Manual Therapy.

[15] Verhagen AP, de Vet HC, de Bie RA, Kessels AG, Boers M, Bouter LM, Knipschild PG (1998). The Delphi list: a criteria list for quality assessment of randomized clinical trials for conducting systematic reviews developed by Delphi consensus. J Clin Epidemiol.

[16] Foley NC, Bhogal SK, Teasell RW, Bureau Y, Speechley MR (2006). Estimates of quality and reliability with the physiotherapy evidence-based database scale to assess the methodology of randomized controlled trials of pharmacological and nonpharmacological interventions. Phys Ther.

[17] Bandeira ADS, Silva KS, Bastos JLD, Silva DAS, Lopes ADS, Barbosa Filho VC (2019). Psychosocial mediators of screen time reduction after an intervention for students from schools in vulnerable areas: a cluster-randomized controlled trial. J Sci Med Sport.

[18] Smith JJ, Morgan PJ, Lonsdale C, Dally K, Plotnikoff RC, Lubans DR (2017). Mediators of change in screen-time in a school-based intervention for adolescent boys: findings from the ATLAS cluster randomized controlled trial. J Behav Med.

[19] Babic M, Lonsdale C, Plotnikoff RC, Eather N, Skinner Lubans GDR (2016). Intervention to reduce recreational screen-time in adolescents: outcomes and mediators from the “Switch-Off 4 Healthy Minds” (S4HM) cluster randomized controlled trial. Prev Med.

[20] Mendoza JA, Baranowski T, Jaramillo S, Fesinmeyer MD, Haaland W, Thompson D, Nicklas TA (2016). Fit 5 kids TV reduction program for latino preschoolers: a cluster randomized controlled trial. Am J Prev Med.

[21] Yilmaz G, Demirli Caylan N, Karacan CD (2015). An intervention to preschool children for reducing screen time: a randomized controlled trial. Child Care Health Dev.

[22] Andrade S, Verloigne M, Cardon G, Kolsteren P, Ochoa-Aviles A, Verstraeten R, Lachat C (2015). School-based intervention on healthy behaviour among Ecuadorian adolescents: effect of a cluster-randomized controlled trial on screen-time. BMC Public Health.

[23] Lubans DR, Smith JJ, Skinner G, Morgan PJ (2014). Development and implementation of a smartphone application to promote physical activity and reduce screen-time in adolescent boys. Front Public Health.

[24] Maddison R, Marsh S, Foley L, Epstein LH, Olds T, Dewes O, Mhurchu CN (2014). Screen-time weight-loss intervention targeting children at home (SWITCH): a randomized controlled trial. Int J Behav Nutr Phys Act.

[25] Hesketh K, Salmon J, Crawford D, Ball K, Abbott G, Campbell K (2014). Impacts of the Melbourne InFANT Program help explain the mechanisms of behaviour change observed in toddlers’ television viewing. J Sci Med Sport.

[26] Birken CS, Maguire J, Mekky M, Manlhiot C, Beck CE, Degroot J, Parkin PC (2012). Office-based randomized controlled trial to reduce screen time in preschool children. Pediatrics.

[27] Mendelsohn AL, Dreyer BP, Brockmeyer CA, Berkule-Silberman SB, Huberman HS, Tomopoulos S (2011). Randomized controlled trial of primary care pediatric parenting programs: effect on reduced media exposure in infants, mediated through enhanced parent-child interaction. Arch Pediatr Adolesc Med.

[28] Leung MM, Agaronov A, Grytsenko K, Yeh M-C (2012). Intervening to reduce sedentary behaviors and childhood obesity among school-age youth: a systematic review of randomized trials. J Obes.

